# A20 Deficiency in Lung Epithelial Cells Protects against Influenza A Virus Infection

**DOI:** 10.1371/journal.ppat.1005410

**Published:** 2016-01-27

**Authors:** Jonathan Maelfait, Kenny Roose, Lars Vereecke, Conor Mc Guire, Mozes Sze, Martijn J. Schuijs, Monique Willart, Lorena Itati Ibañez, Hamida Hammad, Bart N. Lambrecht, Rudi Beyaert, Xavier Saelens, Geert van Loo

**Affiliations:** 1 Inflammation Research Center, VIB, Ghent, Belgium; 2 Department of Biomedical Molecular Biology, Ghent University, Ghent, Belgium; 3 Medical Biotechnology Center, VIB, Ghent, Belgium; 4 Department of Biochemistry and Microbiology, Ghent University, Ghent, Belgium; 5 Department of Respiratory Medicine, Ghent University, Ghent, Belgium; Mount Sinai School of Medicine, UNITED STATES

## Abstract

A20 negatively regulates multiple inflammatory signalling pathways. We here addressed the role of A20 in club cells (also known as Clara cells) of the bronchial epithelium in their response to influenza A virus infection. Club cells provide a niche for influenza virus replication, but little is known about the functions of these cells in antiviral immunity. Using airway epithelial cell-specific A20 knockout (A20^AEC-KO^) mice, we show that A20 in club cells critically controls innate immune responses upon TNF or double stranded RNA stimulation. Surprisingly, A20^AEC-KO^ mice are better protected against influenza A virus challenge than their wild type littermates. This phenotype is not due to decreased viral replication. Instead host innate and adaptive immune responses and lung damage are reduced in A20^AEC-KO^ mice. These attenuated responses correlate with a dampened cytotoxic T cell (CTL) response at later stages during infection, indicating that A20^AEC-KO^ mice are better equipped to tolerate Influenza A virus infection. Expression of the chemokine CCL2 (also named MCP-1) is particularly suppressed in the lungs of A20^AEC-KO^ mice during later stages of infection. When A20^AEC-KO^ mice were treated with recombinant CCL2 the protective effect was abrogated demonstrating the crucial contribution of this chemokine to the protection of A20^AEC-KO^ mice to Influenza A virus infection. Taken together, we propose a mechanism of action by which A20 expression in club cells controls inflammation and antiviral CTL responses in response to influenza virus infection.

## Author Summary

Influenza viruses are a major public health threat. Each year, the typical seasonal flu epidemic affects millions of people with sometimes fatal outcomes, especially in high risk groups such as young children and elderly. The sporadic pandemic outbreaks can have even more disastrous consequences. The protein A20 is an important negative regulator of antiviral immune responses. We show that the specific deletion of *A20* in bronchial epithelial cells improves the protection against influenza virus infections. This increased protection correlates with a dampened pulmonary cytotoxic T cell response and a strongly suppressed expression of the chemokine CCL2 during later stages of infection.

## Introduction

Disease outcome upon exposure to a certain pathogen relies on the capacity of the host to resist and tolerate the infection [1]. Resistance protects the host by suppressing pathogen replication and promoting clearance of the pathogen, a process that is mostly mediated by the innate and adaptive immune system. Tolerance refers to the ability to improve disease outcome without affecting pathogen burden and by limiting tissue damage. An overactive immune response can negatively impact on the disease by causing severe tissue damage [2]. Immunopathology is an important contributor to death during exposure to highly virulent strains of influenza A such as the 1918 H1N1 virus or highly pathogenic avian H5N1 and H7N1 viruses. The mechanisms contributing to immune pathology during flu virus infection have been well documented, and both innate and adaptive immunity seems to be involved [3–6]. However, the exact molecular mechanisms regulating these processes are not well understood.

Detection of Influenza A by the innate immune system occurs by at least three different mechanisms [7]. Firstly, the cytosolic receptor RIG-I detects 5’-triphosporylated influenza virus genome segments [8,9]. In the absence of the viral non-structural protein 1 (NS1), RIG-I induces a strong antiviral type-I interferon response [10]. Secondly, Toll-like receptors such as TLR3 and TLR7 detect virus-associated RNA molecules. TLR7 is mainly employed by IFN producing plasmacytoid dendritic cells, which produce large amounts of type-I IFN upon infection with influenza virus [11,12]. TLR3, which recognizes double stranded RNA of yet undefined origin, has been shown to influence disease outcome following influenza virus infection [13–16]. Thirdly, the NOD-like receptor family member NLRP3 senses multiple influenza virus-associated stimuli, including increased acidification of the cytoplasm mediated by the viroporin M2, leading to the activation of caspase 1 and the release of the cytokines interleukin-1β (IL-1β) and IL-18 [17–19].

A20 (TNF alpha-induced protein 3 or TNFAIP3) is a key player in the termination of inflammation, and has been shown to regulate these innate signalling pathways [19–22]. We previously showed that A20 in macrophages critically suppresses influenza virus-induced innate immune responses and mice deficient in A20 in myeloid cells are protected against influenza A virus infection. This protective effect is mediated by an enhanced innate immune response and a better clearance of the virus [21].

Epithelial cells of the respiratory epithelium are the primary target cells of human influenza viruses and main producers of infectious viral progeny [23]. Very little is known about the physiological contribution of these cells to antiviral immunity. Epithelial cells have long been considered as passive mediators in immunity, functioning primarily as physico-chemical barriers preventing invading pathogens from entering the submucosal layers or the respiratory system. It has become increasingly evident that epithelial cells also maintain important effector functions directing both innate and adaptive immunity crucial for efficient antiviral responses [24]. Respiratory epithelium actively contributes to pulmonary homeostasis [25], immunity against viruses [26] and influenza induced immunopathology [27]. To study how A20 in epithelial cells influences influenza A disease progression, we generated mice lacking A20 specifically in bronchial epithelial cells (also known as club cells or Clara cells). We found that these mice are protected against influenza A virus infection. This protection does not result from an improved viral clearance or increased immune resistance to the virus, but correlates with a dampened pulmonary CTL response and a strongly suppressed expression of the chemokine CCL2 during later stages of infection.

## Results

### A20 regulates inflammatory responses in club cells of the airway epithelium

We studied the role of A20 in airway epithelial cells by crossing conditional *A20* knockout mice (*A20* ^*FL/FL*^, S1A Fig) [28] with double transgenic animals carrying a reverse tetracycline transactivator controlled by the rat CCSP promoter (CCSP-rTA) and a Cre recombinase under control of the (TetO)_7_CMV operator [29,30]. This generated *A20* ^*FL/FL*^/CCSP-rTA/(tetO)_7_-Cre triple transgenic offspring, hereafter referred to as A20^AEC-KO^ mice. Treatment of these mice with doxycycline enabled temporally controlled inactivation of the *A20* gene specifically in club cells (also known as Clara cells) [31], which constitute most of the epithelial cells found in the proximal airways of the mouse respiratory tract [32]. To ensure deletion of *A20* in club cells at all times, breeding pairs and offspring were continuously maintained on a doxycycline diet. A20^AEC-KO^ mice were born at Mendelian ratios and displayed no developmental defects or signs of spontaneous pulmonary inflammation.

Southern blot analysis on genomic DNA extracted from total lungs of A20^AEC-KO^ mice and control wild-type (A20^WT^) littermates showed Cre-mediated recombination of the *A20* ^*FL/FL*^ allele only in lungs, but not in spleens, of A20^AEC-KO^ mice (S1B Fig). Since club cells represent a minor fraction of total lung tissue, only partial recombination was observed in total lung tissue from A20^AEC-KO^ mice. Semi-quantitative PCR analysis on genomic DNA isolated from various tissues of A20^AEC-KO^ mice, confirmed the deletion of *A20* specifically in lung tissue (S1C Fig). As A20 is expressed at low levels in most cell types including airway epithelial cells, we induced its expression by intratracheal instillation of LPS, a TLR4 agonist and known inducer of A20 in lung epithelium [33]. Analysis of A20 expression of Club cells purified from the lungs of A20^AEC-KO^ mice by Western blot showed the absence of A20 at the protein level (S1D Fig). Absence of A20 was finally confirmed by immunohistochemistry on lung tissue isolated from A20^AEC-KO^ mice (S1E Fig).

**Fig 1:**
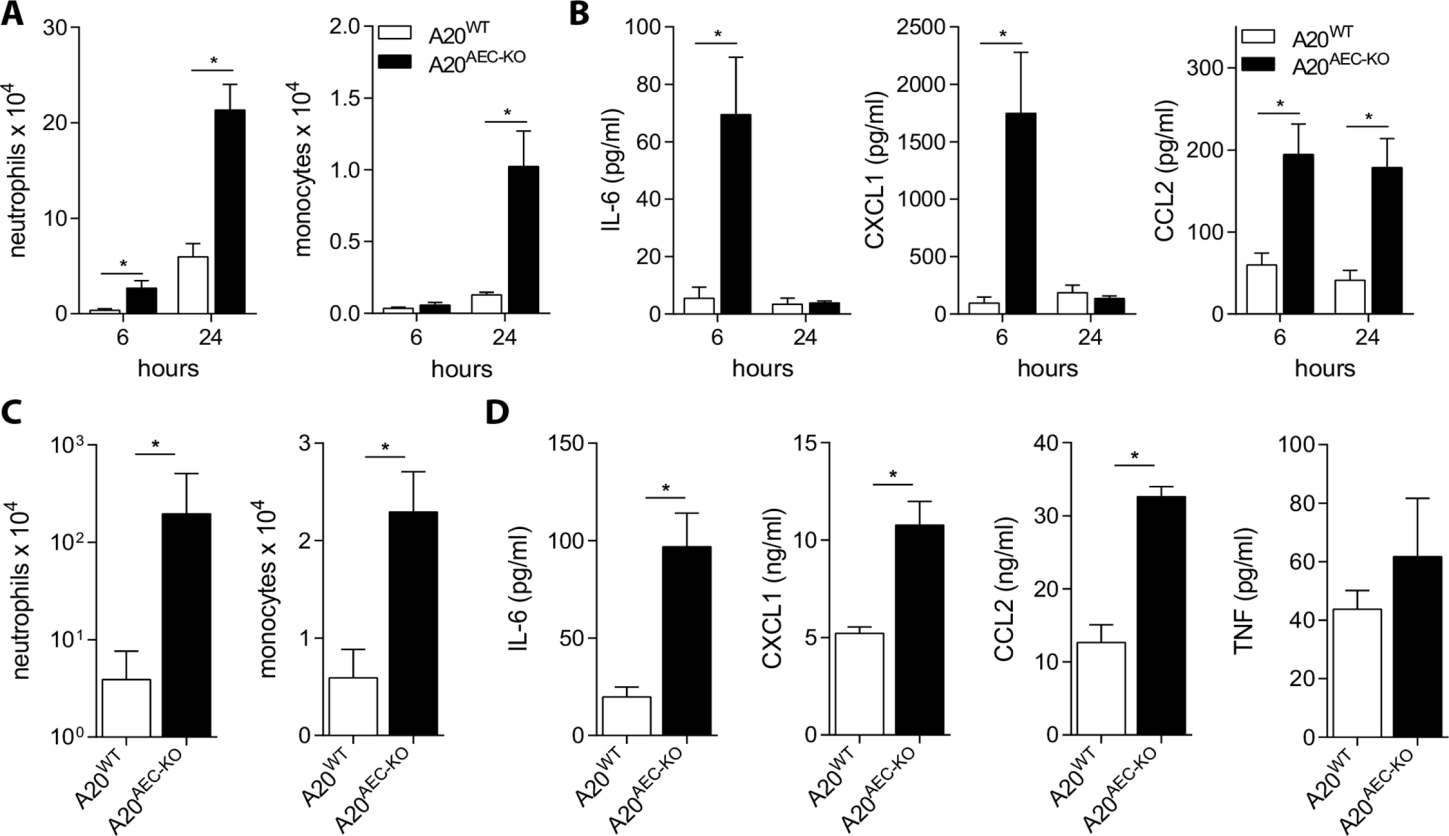
A20 expression in club cells controls TNF and poly(I:C) induced inflammation. Intratracheal administration of 0.5μg TNF (A and B) or 50μg poly(I:C) (C and D) to A20^AEC-KO^ or wild type littermates (A20^WT^). Absolute numbers of neutrophils or monocytes in bronchoalveolar lavages (BAL) as determined by flow cytometry at 6h and 24h post-treatment for TNF (A) or 24h post-treatment for poly(I:C) (C). IL-6, CXCL1 (KC), CCL2 (MCP-1) and TNF [only for poly(I:C)] protein levels in BAL fluid detected by Multiplex immunoassay (B and D). Data represent mean ± SEM of at least 4 mice per group (*p < 0.05; Student’s *t*-test).

A20 is a negative regulator of multiple signalling pathways induced by inflammatory stimuli including TNF and TLR ligands [34,35]. We tested if A20 also regulates these pathways in club cells by intratracheal administration of TNF and poly(I:C), an agonist of the TLR3, RIG-I and MDA5 signalling receptors [36,37]. We found increased numbers of neutrophils and monocytes recruited into the bronchoalveolar space of A20^AEC-KO^ mice compared to control littermates upon engagement of these receptors (Fig 1A and 1C). Also significantly higher levels of IL-6, CXCL1 (KC), CCL2 (MCP-1), but not TNF were detected in the bronchoalveolar lavage (BAL) fluid of mice lacking A20 in club cells (Fig 1B and 1D). These results demonstrate that A20 expression in club cells negatively controls inflammatory responses following exposure of the airway to TNF and poly(I:C). These data are in accordance with previously published results showing a similar regulatory role for A20 in various other cell types [34].

### A20^AEC-KO^ mice tolerate infections with influenza A virus

**Fig 2:**
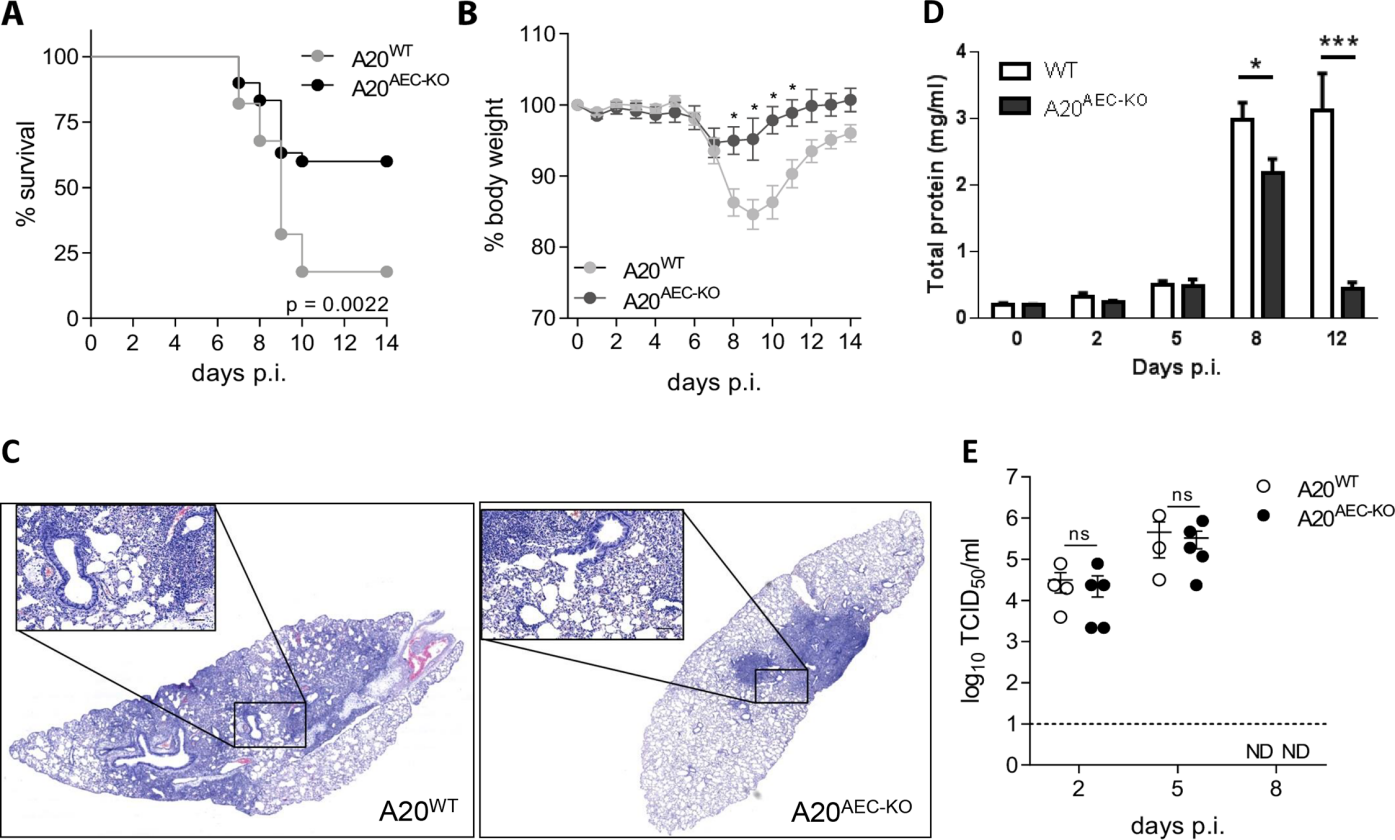
Deficiency of A20 in club cells protects against Influenza A infection. (A) Survival of A20^AEC-KO^ (n = 30) or wild type littermates (A20^WT^, n = 28) infected with a lethal dose of Influenza A X-47 (2 X LD_50_). (B) Weight loss of A20^AEC-KO^ (n = 6) or wild type littermates (A20^WT^, n = 8) monitored until 14 days post infection (days p.i.) upon infection with a sublethal dose of X-47 (0.05 X LD_50_). (C) Representative pictures from hematoxylin and eosin stained lung tissue sections from A20^AEC-KO^ and control wild-type (WT) littermate mice at day 8 p.i. Detail, scale bar 100 μm (D) Total protein concentration using Bradford assay in BAL fluid of A20^AEC-KO^ and control A20^AEC-WT^ littermates at different time points after sublethal IAV infection (n = between 3 and 11 for each time point). *p < 0.05; ***p<0.001. (E) Pulmonary viral titers determined by TCID_50_ at 2, 5 and 8 days p.i. after infection with a sublethal dose (0.05 X LD_50_) of X-47. Dashed line represents detection limit (ND = not detected). Data were pooled from 3 independent experiments and analyzed using Log-rank (A). Data are representative of at least 2 independent experiments and were analysed using 2-way ANOVA (B, *p < 0.05) or Student’s *t*-test (C, ns = not significant) and are shown as mean ± SEM.

Since poly(I:C) is often used as a stimulus that mimics RNA virus infection, we next investigated the *in vivo* role of A20 in club cells in a mouse model of influenza A virus (IAV) infection. We used the H3N2 mouse adapted influenza A strain X-47 in most of the experiments [38]. Immunohistochemical staining of the club cell marker CCSP and the influenza A ion channel protein M2, revealed that X-47 infected CCSP expressing club cells, along with alveolar epithelial cells (S2A and S2B Fig). This allowed us to directly study the *in vivo* role of A20 in the primary target cells of influenza A virus. A20^AEC-KO^ had a clear survival advantage compared to their control littermates following infection with a dose of X-47 virus that proved lethal for most wild type mice (Fig 2A). Upon infection with a sublethal dose of X-47, A20^AEC-KO^ mice displayed less morbidity and weight loss at later stages (> 7 days) of infection (Fig 2B). Challenge with A/Puerto Rico/8/34 (PR8, H1N1 subtype) influenza virus confirmed the reduced susceptibility of A20^AEC-KO^ mice to IAV infection (S3 Fig). This difference was also evident by histological analysis of lung tissue from X-47-infected mice (Fig 2C and S4A Fig) and by quantification of the total protein content in BAL fluid as a measure of lung damage and vascular leakage (Fig 2D). We did not detect significant differences in viral titres in the lungs of A20^AEC-KO^ and A20^WT^ mice at day 2 and day 5 post-infection (Fig 2E). These data indicate that the disease protection in A20^AEC-KO^ mice is likely not a consequence of an enhanced intrinsic capacity of A20 deficient club cells to inhibit viral replication. The levels of IFNα and IFNβ, cytokines with potent antiviral activity, and the IFN-inducible chemokine CXCL10, were comparable in BAL fluid isolated from A20^AEC-KO^ and A20^WT^ animals at different time points post-infection, suggesting that the type-I IFN response is not differentially regulated (S4B Fig). More importantly, we could not detect virus in the lungs of both A20^AEC-KO^ and A20^WT^ mice at day 8 post infection (Fig 2E), which is the time during infection where both groups of mice differ in morbidity. Finally, since A20 is known to confer protection against cell death in multiple cell types (Catrysse et al., Trends Immunol., 2014), we assessed if A20 deficiency in club cells could sensitize these cells to apoptosis after IAV infection. In none of the assays performed, however, we could detect any significant difference in cell death between A20^AEC-KO^ lung samples and control samples (S5 Fig).

The absence of A20 in club cells thus significantly improves disease outcome upon influenza A virus infection without altering viral clearance or type-I IFN responses. These data indicate that A20^AEC-KO^ mice are protected from influenza A virus infection by a mechanism that involves increased tolerance, rather than increased antiviral resistance.

### The pulmonary CTL response is dampened in A20^AEC-KO^ mice

**Fig 3:**
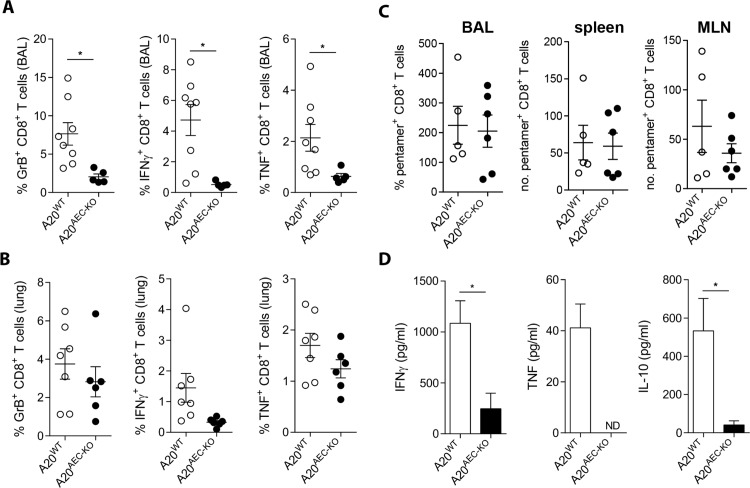
The antiviral CTL response is attenuated in A20^AEC-KO^ mice. (A and B) *In vivo* intracellular staining for Granzyme B (GrB), IFNγ, and TNF on activated (CD62L^lo^) CD8^+^ T cells from BAL (A) and lungs (B) of A20^AEC-KO^ or A20^WT^ mice infected with 0.05 X LD_50_ X-47 at day 8 post infection. (C) NP-specific pentamer staining of CD8^+^ T cells in BAL, spleens and mediastinal lymph nodes (MLN) of mice infected with 0.05 X LD_50_ X-47 measured at day 8 (D) IFNγ, TNF and IL-10 protein levels measured by ELISA on BAL fluid collected from A20^AEC-KO^ or A20^WT^ mice infected with 0.05X LD_50_ X-47 at day 8 post infection. Data are representative of 2 independent experiments and show as mean ± SEM of at least 6 mice per group (*p < 0.05; Student’s *t*-test).

The late onset (> 7 days) of protection to influenza A of A20^AEC-KO^ mice suggests that the effect is driven by the adaptive immune system. Although viral clearance and host survival critically depend on the recruitment of virus-specific CD8^+^ cytotoxic T cells (CTL) to the lung, influenza A-associated pulmonary immunopathology can be inflicted by an excessive antiviral CTL response. CTLs are major producers of TNF during influenza A virus infection which is known to contribute to immunopathology [5,39,40]. Analysis of CD8^+^ T cells by *in vivo* intracellular cytokine staining showed that A20^AEC-KO^ mice displayed reduced numbers of Granzyme B, IFNγ and TNF expressing activated (CD62L^lo^) CD8^+^ T cells in the brochoalveolar space and to a lesser extend in the lung tissue at day 8 post infection with X-47 virus (Fig 3A and 3B). NP-pentamer staining of CD8^+^ T cells from the mediastinal lymph node, spleen and BAL revealed that there was no significant difference between control and A20^AEC-KO^ mice (Fig 3C). This suggests that the extent of influenza antigen-specific CD8^+^ T cell priming is comparable in the two mouse strains but that their activation in the lung compartment is different. A20^AEC-KO^ mice also showed reduced levels of IFNγ and TNF protein levels in the BAL fluid (Fig 3D). Upon entering the lung, effector CD8^+^ T cells are programmed to produce high levels of the anti-inflammatory cytokine IL-10, which is an important mechanism to reduce immunopathology [41]. Measurements of IL-10 in BAL fluid showed that also IL-10 levels are lower in A20^AEC-KO^ mice (Fig 3D). This indicates that the reduced CTL response in A20^AEC-KO^ mice is caused by a local signal from the pulmonary environment instead of a skewing of CD8^+^ T cells towards an anti-inflammatory phenotype.

A decreased CTL response can be the result of less effective priming of naïve T cells in the lung draining lymph nodes. Activated antigen-loaded dendritic cells travelling from the lung to the lung draining lymph nodes dictate the outcome of the CTL response [42]. However, we found no significant differences in the accumulation of CD11b^-^, CD11b^+^ or inflammatory dendritic cells (iDC) in the lung draining mediastinal lymph nodes of A20^AEC-KO^ mice compared to control mice (S6A Fig). Furthermore, *in vitro* re-stimulation with NP peptide of single cell suspensions prepared from spleens of A20^WT^ and A20^AEC-KO^ mice infected with X-47 showed similar numbers of NP-specific CD8^+^ T cells as measured by IFNγ producing activated CD8^+^ T cells. In contrast, based on this restimulation assay, less NP-specific T cells responded to peptide re-stimulation by inducing IFNγ expression in the lungs of A20^AEC-KO^ mice (S6B Fig). Loss of A20 in club cells did not affect antiviral humoral immunity as no significant differences in the levels of X-47 neutralizing antibodies were observed in sera from challenged A20^AEC-KO^ mice compared to wild-type mice (S6C Fig).

Together, these data indicate that selective deletion of A20 in club cells suppresses the pulmonary CTL response against influenza A virus leading to a decreased production of cytotoxic CTL effector cytokines such as TNF.

### CCL2 controls tolerance of A20^AEC-KO^ mice to influenza A infection

**Fig 4:**
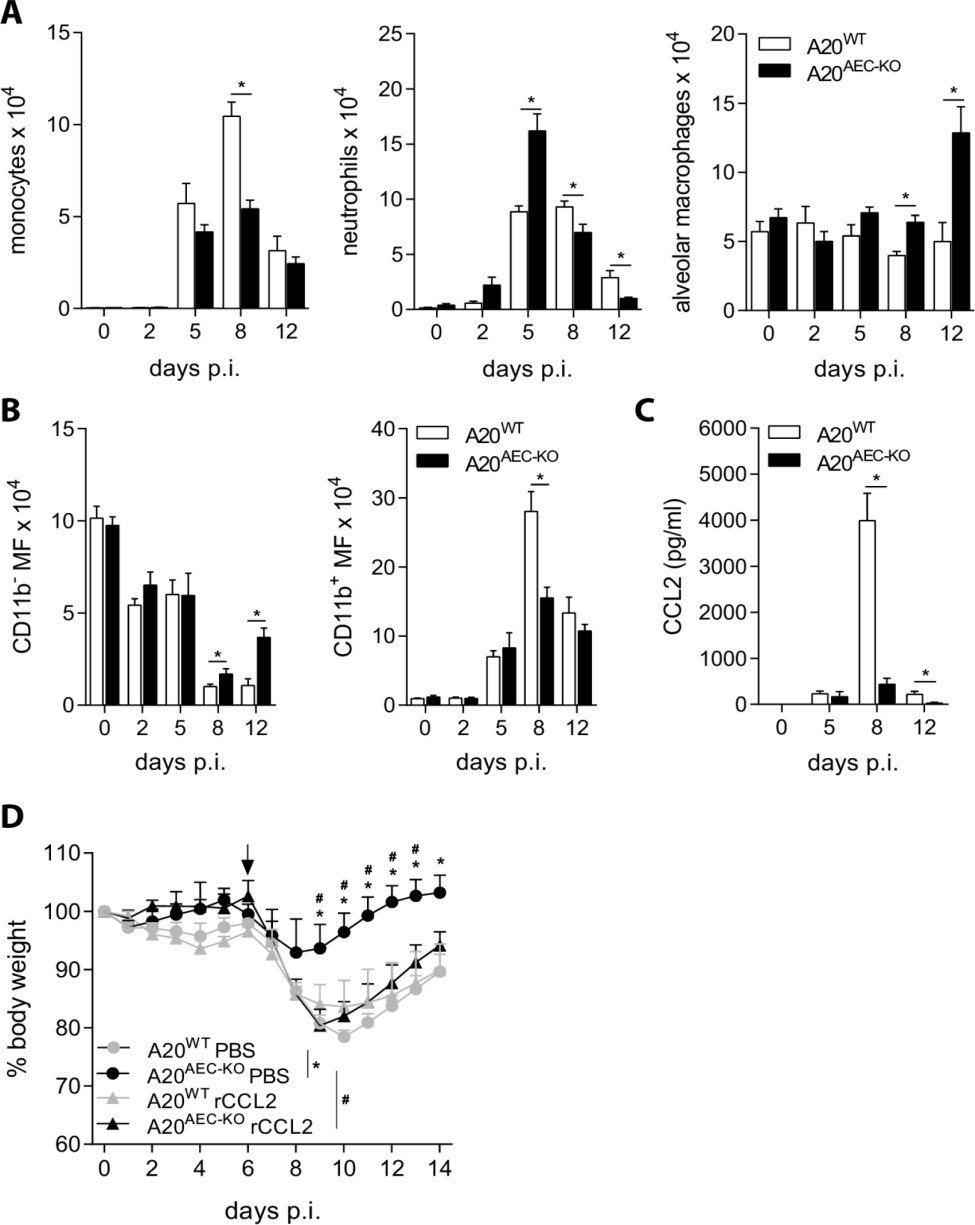
Decreased CCL2 levels protect A20^AEC-KO^ mice against influenza A infection (A) Absolute numbers of monocytes, neutrophils and alveolar macrophages in bronchoalveolar lavages (BAL) of A20^AEC-KO^ or A20^WT^ mice at 2, 5, 8 and 12 days post-infection (days p.i.) with 0.05 X LD_50_ X-47. (B) Absolute numbers of resident CD11b^-^ or recruited CD11b^+^ macrophages in the lungs of A20^WT^ and A20^AEC-KO^ mice. (C) CCL2 (MCP-1) protein levels in BAL fluid measured by Multiplex immunoassay at indicated time points post-infection. (D) Weight loss of A20^AEC-KO^ and A20^WT^ mice infected with 0.05 X LD_50_ X-47. At day 6 p.i. (indicated by an arrow) mice received intranasal treatment with 50 μg/kg recombinant CCL2 (rCCL2) or PBS. Data were analysed using Student’s *t*-test (A, B and C *p < 0.05) and 2-way ANOVA (D, *p < 0.05 for A20^AEC-KO^ PBS vs. A20^WT^ PBS and ^#^p < 0.05 for A20^AEC-KO^ PBS vs A20^AEC-Cre^ rCCL2). Data represent mean ± SEM of at least 3 mice per group. Data are representative of at least 2 independent experiments.

We next addressed whether A20 deficiency in club cells and associated suppression of pulmonary CTL responses affects the recruitment of innate immune cells to the lungs. The number of recruited monocytes, neutrophils and macrophages in BAL was measured at different time points after infection with X-47 virus. At late time points post-infection (> 5 days), the timing at which A20^AEC-KO^ mice show protection from influenza compared to control mice (Fig 2), recruitment of monocytes and neutrophils was significantly lower in A20^AEC-KO^ compared to wild type mice (Fig 4A). CD11b^-^ macrophages (“resident” macrophages) constituted the predominant macrophage population in the lung parenchyma of unchallenged mice and at early time points post-infection, while the number of CD11b^+^ macrophages (“recruited” macrophages) increased, starting around day 5 and peaking around day 8 post-infection (Fig 4B). The lungs of A20^AEC-KO^ mice contained more CD11b^-^ resident macrophages and alveolar macrophages compared to control littermates at peak CTL response, 8 days post-infection, and this difference sustained at later time points (Fig 4A and 4B).

Recruitment of macrophages to lungs is dependent on the chemokine CCL2 (MCP-1) [43–45]. In agreement with the elevated levels of recruited monocytes and macrophages at day 8 post-infection in control A20^WT^ mice, higher levels of CCL2 could be detected in BAL fluid of these mice compared to A20^AEC-KO^ littermates at this stage (Figs 4C and S7A). Other chemokines, such as KC (CXCL1) and Rantes (CCL5), were also differentially expressed in A20^AEC-KO^ and control A20^WT^ mice at day 8 post-infection, although at lower levels (S7A Fig). Immunohistochemical analysis of X-47-infected lung tissue confirmed enhanced CCL2 staining in CCSP^+^ lung epithelial cells from A20^WT^ mice compared to A20^AEC-KO^ mice (S7B Fig). To assess the importance of reduced CCL2 levels in A20^AEC-KO^ mice for their protective phenotype upon influenza virus infection, we administered recombinant mouse CCL2 (rCCL2) to A20^AEC-KO^ mice at day 6 post-infection. In contrast to control (PBS) treated A20^AEC-KO^ mice, which again show protection from the virus challenge compared to wild-type littermates, rCCL2 treated A20^AEC-KO^ littermates mice are sensitized to infection and no longer show differences compared to PBS or cCCL2 treated wild-type mice (Fig 4D). Together, these results show that the protection of A20^AEC-KO^ mice from infection results from a reduced CCL2-dependent recruitment of innate immune cells to the lungs of infected mice.

## Discussion

A20 is an essential negative regulator of NF-κB signaling, and A20 deficient mice die prematurely due to massive multi-organ inflammation triggered by infiltrating intestinal bacteria [46,47]. We showed in this study that specific deletion of A20 in respiratory epithelial cells protects mice from Influenza A virus-induced morbidity and lethality. Viral clearance and the production of the antiviral cytokines IFNα and IFNβ was similar in A20^AEC-KO^ and wild-type mice, in agreement with literature stating that epithelial cells are not the primary producers of type-I IFNs upon respiratory virus infection [48]. Interestingly, although the initial recruitment of innate cells and CTLs into the lungs of A20^AEC-KO^ mice was sufficient to clear the virus by 8 days post infection, monocyte recruitment and the local CTL response in the lung were markedly reduced during later stages of infection. This was rather surprising since A20 is characterized as a negative regulator of the antiviral immune response [49]. Indeed we could confirm such a role for A20 in a surrogate viral infection model using intratracheal instillation of the double stranded RNA mimic poly(I:C) showing hyperactive immune responses in AEC-specific A20 knockout mice.

Protection of A20^AEC-KO^ mice against influenza virus infection correlated with reduced recruitment of monocytes and CD11b^+^ macrophages to the lungs. In line with this, the levels of the monocyte-recruiting chemokine CCL2 were much lower in lungs of A20^AEC-KO^ mice than in wild type mice on days eight and twelve after infection. It has been reported that administration of an MCP-1/CCL2 blocking antibody can reduce mortality and morbidity following influenza A virus infection [50]. Increased CCL2 levels have been reported in patients that had been infected with H7N9 virus [51]. CCL2 contributes to tissue immunopathology following influenza virus infection by its pro-inflammatory effects on macrophages and monocytes [52]. Recruitment of inflammatory macrophages in a manner dependent on the CCL2 receptor CCR2 contributes substantially to lung damage [53]. In line with this, we found that intranasal instillation of CCL2 in A20^AEC-KO^ mice rendered these mice as susceptible to disease caused by influenza A virus as wild type mice. In aggregate, the reduced levels of CCL2 in A20^AEC-KO^ mice could explain their increased tolerance to the infection. Yet after exposure of A20^AEC-KO^ mice to double stranded RNA, a surrogate viral PAMP, we observed increased levels of CCL2 compared to wild type controls. The latter observation is in line with the regulation of CCL2 expression by NF-κB [54]. So what caused the reduced levels of CCL2 in the A20^AEC-KO^ mice at later time points after influenza virus infection? It was remarkable that reduced disease, lower CCL2 levels and decreased CD8^+^ T cell activation coincided. CCL2 expression can be induced by IFNγ [55]. Therefore, the reduced numbers of IFNγ-producing CD8^+^ T cells could explain the lower CCL2 levels and monocyte infiltration in the A20^AEC-KO^ mice. Similarly trans-presentation of TNF by influenza HA specific CD8^+^ T cells to lung epithelial cells has been shown to induce strong expression of CCL2 leading to extensive lung injury due to infiltrating monocytes. Increased CCL2 release by club cells might in addition recruit monocyte-derived inflammatory dendritic cells into the lungs, which could sustain local CTL responses and inflicting further damage to the lung [56,57]. Pro-inflammatory cytokine expression by club cells contributes to immunopathology during later stages of influenza infection [27]. Our study shows that expression of A20 in these cells might prevent lung damage by the host’s immune system. The exact molecular mechanism of how the specific deletion of A20 in club cells leads to increased tolerance to influenza infection is not understood at this moment and is subject for future research.

In conclusion, these data show that loss of A20 in respiratory epithelium can protect mice following Influenza A virus infection. In agreement with previous results, showing that deletion of A20 in myeloid cells also protects from Influenza [21], these data suggest that inhibiting A20 expression, for example by local administration of interfering RNAs, might be promising as a new therapeutic strategy to control disease caused by influenza A virus infection.

## Materials and Methods

### Ethics statement

All experiments on mice were conducted according to the national (Belgian Law 14/08/1986 and 22/12/2003, Belgian Royal Decree 06/04/2010) and European (EU Directives 2010/63/EU, 86/609/EEG) animal regulations. Animal protocols were approved by the Ethics Committee of Ghent University (permit number LA1400091, approval ID 2010/001). All efforts were made to reduce suffering of animals. Before procedures mice were anesthetised by intraperitoneal (i.p.) injection of ketamine and xylazin.

### Mice

Conditional *A20* knockout mice harbouring two *LoxP* sequences flanking exon 4 and exon 5 (*A20* ^*FL/FL*^) were generated as previously described [28] and were crossed with CCSP-rTA/(tetO)_7_-Cre mice to specifically delete A20 in airway epithelial cells (AEC) (provided by Dr. J. Whitsett, Cincinnati Children’s Hospital, USA) [29,58]. *A20* ^*FL/FL*^/CCSP-rTA/(tetO)_7_-Cre triple transgenic offspring were fed doxycycline-containing food pellets to delete *A20* expression (625 mg/kg, Special Diet Services). All experiments were performed on age- and sex- matched littermates. All mice used in this study carried the *A20* ^*FL/FL*^ and CCSP-rTA alleles. Those expressing the (TetO)_7_ allele were termed A20^AEC-KO^ and those lacking this allele were considered as wild type controls (A20^WT^). Mice were housed in individually ventilated cages at the VIB-UGent Inflammation Research Center (IRC) in a specific pathogen-free animal facility.

### Poly (I:C) and TNF administration

After anesthetisation, mice received an intratracheal dose of 50 μg low molecular weight endotoxin-free poly(I:C) (Invivogen) or 0.5 μg recombinant mouse TNF (in house production) in 50 μl PBS. Six or 24 h after instillation mice were sacrificed and broncheoalveolar lavages (BAL) were collected for flow cytometric analysis and cytokine / chemokine analysis.

### Viruses

Mouse adapted influenza A X-47 (H3N2) and PR8 (H1N1) were propagated in MDCK (Madin-Darby canine kidney, ATCC) cells. After anesthetisation, mice were infected intranasally with X-47diluted in 50 μl PBS. For lethal and sublethal infections, mice received 2 X LD_50_ X-47 and 0.05 X LD_50_ X-47 or 0.17 X LD_50_ PR/8, respectively. Mice were euthanized when weight loss exceeded 25% of the initial body weight. Recombinant mouse CCL2 (R&D Systems, endotoxin levels <0.01 EU per μg of protein as measured by the LAL method) was administered intranasally at a dose of 50 μg/kg at day 6 post infection. Pulmonary viral titres were determined by median tissue culture infectious dose (TCID_50_) determination using MDCK cells. Lungs were homogenized in PBS using a Polytron homogenizer (Kinematica) and ten-fold serial dilutions of cleared lung homogenates were incubated on MDCK cells in DMEM supplemented with trypsin (1 μg/ml), 2 mM L-glutamine, 0.4 mM sodium pyruvate and antibiotics. After 5 days, 0.5% chicken red blood cells (RBC) were added to cell culture supernatant and end-point dilution hemagglutination was monitored. TCID_50_ titres were calculated according to the method of Reed and Muench [59]. HAI titres in serum of infected mice were determined as follows: serum was treated for 18 h at 37°C with receptor-destroying enzyme (RDE/Cholera filtrate, Sigma Aldrich) to remove sialic acids from serum proteins and prevent nonspecific inhibition of agglutination. RDE was then inactivated by the addition of 0.75% sodium citrate and heating at 56°C for 30 min. To remove sialic acid binding proteins, sera were cleared with 1/10 volume 50% chicken RBC. Titration was done by incubating a two-fold dilution series of sera with 4 HA units of X-47 for 1 h in 96-well U-bottom plate. An equal volume of 0.5% chicken RBC was then added and HAI titres were read after 30 min.

### Club cell purification

The method for club cell isolation was adapted from [60]. Lungs were inflated with 0.25% trypsin/HBSS and incubated in DMEM at 37°C for 20 min. After trypsin neutralisation with FCS, lungs were cut into small 1 mm^3^ sections and sequentially filtered without homogenisation through a 40 μm cell strainer. The cells were then placed into a humidified incubator at 37°C for 2 h to remove adherent cell populations (macrophages and fibroblasts). Club cells were specifically pelleted by centrifugation at 30 g for 8 min.

### Flow cytometry

Lungs and mediastinal lymph nodes (MLN) were dissected into small 1 mm^3^ sections and incubated with collagenase type IV (1 mg/ml, Worthington) and DNase I (100 U/ml, Roche) at 37°C for 30 min. Subsequently, samples were filtered and homogenised through a 40 μm cell strainer. For BAL, tracheas were cannulated and lungs were flushed 4 times with HBSS with 1 mM EDTA. The first ml was treated with EDTA-free protease inhibitor tablets (Roche) and frozen separately for cytokine/chemokine analysis. After treatment with red blood cell lysis buffer (Sigma Aldrich), cells were stained with monoclonal antibodies directed against MHC-II (I-A/I-E) (M5/114.15.2), CD11c (N418) CD8α (53–6.7), F4/80 (BM8), CD62L (MEL-14), Granzyme B (NGZB) B220 (RA3-6B2) from eBiosciences and CD45 (30-F11), CD3ε (500A2), Gr1 (RB6-8C5), Ly6G (1A8), Ly6C (AL-21) CD11c (HL3), CD11b (M1/70), CD4 (GK1.5), CD8α (53–6.7), IFNγ (XMG1.2), TNF (MP6-XT22) and CD16/32 (2.4G2) from BD Pharmingen. Samples were acquired on a LSRII Cytometer (BD Biosciences) and analysis was performed using FACSDiva software (BD Biosciences).

### Immunohistochemistry

Lungs were inflated with 4% paraformaldehyde. After 1 hour lungs were washed in PBS and embedded in paraffin. 5 μm thick tissue sections were cut from paraffin blocks. For immunohistochemistry, sections were dewaxed, dehydrated and incubated in Dako antigen retrieval solution, brought to boiling temperature and allowed to cool down for 2 hours. Endogenous peroxidase activity was blocked by immersing slides in 3% H_2_O_2_ for 5 min. Sections were blocked and permeabilized in 10% goat serum and 1% Triton X-100. Sections were incubated overnight at 4°C in blocking buffer with anti-M2 (in house preparation) and anti-CCSP (Abcam) antibody. Subsequently, slides were incubated with secondary antibody for 1 h (polymer HRP-labelled anti-mouse/rabbit/rat; Vector Laboratories) and peroxidase was detected by diaminobuteric acid (DAB) substrate (Sigma Aldrich). Tissue sections were counterstained with Mayer’s haematoxylin, rehydrated and mounted in Pertex mounting medium (Histolab). For A20 staining, sections were incubated for 60 min at 4°C in blocking buffer with anti-A20 (ProSci) antibody, and subsequently with HRP-labeled anti-rabbit IgG and FITC-labeled anti-HRP. For CCL2/MCP1 staining, sections were incubated for 30 min with anti-CCL2 (Abcam, clone ECE.2) antibody, followed by Cy3-labeled anti-rat antibody.

### *In vitro* and *in vivo* intracellular staining of activated T cells

For *in vitro* peptide stimulation, 5 x 10^6^ cells isolated from spleen or lungs of animals infected with X-47 were stimulated with different concentrations of NP-peptide (ASNENMETM, JPT peptide solutions). After 18h, brefeldin A was added to the cell culture and after 6 h cells were collected and stained for cell surface markers and IFNγ. *In vivo* intracellular staining for GrB, IFNγ and TNF from freshly isolated BAL and lungs was performed as described previously [61]. Mice were treated intranasally with 50 μg brefeldin A (Sigma Aldrich) 6 h before tissue collection and single cell suspensions from BAL and lungs were prepared in the presence of 3 μg/ml brefeldin A. Cells were stained for cell surface markers and intracellular molecules and fixed and permeabilized in Cytofix / Cytoperm (BD biosciences) according to manufacturer’s instructions. Activated CD8^+^ T cells were identified as CD3ε^+^, CD8α^+^ and CD62L^lo^. Live/Dead fixable aqua dead cell stain (Molecular Probes) was used to discriminate live from dead cells.

### Pentamer staining

Spleens, mediastinal lymph nodes and bronchoalveolar lavage (BAL) fluid were isolated from infected and non-infected mice. Single cell suspensions were prepared in PBS containing 0.5% BSA. After removal of red blood cells by osmotic lysis, cells were stained with Aqua Live/Dead (Life Technologies), anti-CD16/CD32 (clone 2.4G2; Fc Block; Becton Dickinson Biosciences), anti-CD19 (clone 1D3; Becton Dickinson Biosciences), anti-CD3ε (clone 17A2; Becton Dickinson Biosciences), anti-CD8α (clone 53–6,7; Becton Dickinson Biosciences) and H-2Db ASNENMETM pentamer (ProImmune) for 25 minutes at 4°C. Prior to measurements, CountBright Absolute Counting Beads were added to each sample. Cells were measured on a BD LSR II cytometer (BD Biosciences) and analysed using FlowJo software (Tree Star).

### Cytokine quantification

Detection of CCL2 (MCP-1), CCL5 (Rantes), CXCL1 (KC), IL-6 and TNF in BAL fluid was performed using Multiplex immunoassay technology (BioRad). IFNγ, IL-10 (eBioscience) IFNβ and IFNα (R&D Systems and Invivogen) protein levels were determined by ELISA.

### Western, Southern blotting and genomic PCR

For Western blotting, cells were lysed at 4°C for 15 min in lysis buffer (200 mM NaCl, 1% NP-40, 10 mM Tris-HCl pH 7.5, 5 mM EDTA and 2 mM DTT) supplemented with protease and phosphatase inhibitors. Cell lysates were subsequently separated by SDS-PAGE and analyzed by western blotting and ECL detection (Perkin Elmer Life Sciences). Immunoblots were revealed with anti-A20 (Santa Cruz), anti-actin (MP Biomedicals) and HRP-linked anti-mouse (GE Healthcare) antibodies. For Southern blotting, genomic DNA was digested with BamHI yielding 6.5- and 13.5-kb fragments for *A20* floxed and deleted alleles, respectively. DNA was separated on an agarose gel and transferred to a nitrocellulose membrane. Hybridization was performed with a ^32^P-labeled probe. *A20* specific genomic PCR was performed using the following primers: 5’-CAC AGA GCC TCA GTA TCA TGT-3’, 5’-CCT GTC AAC ATC TCA GAA GG-3’ and 5’ GCA GCT GGA ATC TCT GAA ATC 3’.

### Cell death measurement

Apoptosis was analysed by fluorescence microscopy using an *in situ* cell death detection kit (Roche). Caspase activity was measured by incubation of 25 μg tissue homogenate with 50 μM acetyl-Asp-Glu-Val-Asp-aminomethylcoumarin (Ac-DEVD-amc) (Peptide Institute, Osaka, Japan) in 150 μl cell-free system buffer (10 mM HEPES–NaOH pH 7.4, 220 mM mannitol, 68 mM sucrose, 2 mM NaCl, 2.5 mM KH_2_PO_4_, 0.5 mM EGTA, 2 mM MgCl_2_, 5 mM pyruvate, 0.1 mM PMSF, 1 mM dithiothreitol). The release of fluorescent 7-amino-4-methylcoumarin was measured for 50 min at 2-min intervals by fluorospectrometry at 360 nm excitation and 480 nm emission wavelength, using a Cytofluor device (PerSeptive Biosystems, Cambridge, MA). The maximal rate of increase in fluorescence was calculated (Δfluorescence/min). Lungs were prepared as described for flow cytometric analysis and cells were stained with antibodies directed against CD326 (EpCAM, eBioscience clone G8.8), CD45 and CD31 (eBioscience, clone 390). Prior to flow cytometric analysis cells were washed and incubated with Annexin V and propidium iodide in Annexin V binding buffer according to manufacturer’s instructions (BD Biosciences).

### Statistics

Data were analysed using GraphPad Prism Software. Results are expressed as the mean ± SEM. Statistical significance between experimental groups was assessed using an unpaired two-sample Student’s *t* test. Statistical significance of differences between survival rates was analysed by comparing Kaplan-Meier curves using the log-rank test.

## Supporting Information

S1 Fig Analysis of *A20* (*Tnfaip3*) deletion in club cells of A20^AEC-KO^ mice. (A) Scheme of conditional *A20* gene targeting. Boxes indicate exons 1–9 (E1-E9; black boxes depict coding sequence). E4 and E5 are flanked by 2 LoxP sites (arrowhead). Restriction enzymes (B, BamHI and V, EcoRV) and the location of the probe for Southern Blot analysis are also annotated. (B) Southern blot on genomic DNA isolated from spleens and lungs of A20^AEC-KO^ or A20^WT^ mice. DNA from heterozygous (A20^+/-^) MEFs was used as a control. (C) A20 specific PCR using primers that discriminate between floxed and deleted A20 alleles on genomic DNA isolated from lungs of A20^WT^ or A20^AEC-KO^ mice or DNA from different organs of A20^AEC-KO^ mice. (D) Western blot for A20 and actin on club cells purified from lungs of A20^WT^ or A20^AEC-KO^ mice after intratracheal instillation of 1 μg LPS for 24 hours. (E) Immunohistological section of A20^WT^ and A20^AEC-KO^ lung tissue stained with anti-A20 antibody (green). Cell nuclei are staining with DAPI. Scale bar 20μm.
(TIF)

S2 Fig Influenza A X-47 infects CCSP expressing club cells and IFNα expression after X-47 challenge. (A) Immunohistochemical stain for CCSP on lung sections of wild type mice (upper scale bar 50μm, lower scale bar 20μm). (B) Immunohistochemical stain for influenza A M2 protein on wild type mice mock (left) or X-47 virus infected (right) analyzed 4 days (4d) after infection (upper scale bar 50μm, lower scale bar 20μm). Lower panels represent magnifications of boxed sections of upper panels. Arrows indicate M2-positive alveolar epithelial cells.
(TIF)

S3 Fig Deficiency of A20 in club cells protects against PR8 IAV infection. Weight loss of A20^AEC-KO^ (n = 6) or wild type littermates (A20^WT^, n = 9) monitored until 14 days post infection (days p.i.) with a sublethal dose of the A/Puerto Rico/8/34 (PR8) strain (0.17 X LD_50_). Data were analysed using 2-way ANOVA (*p < 0.05) and are shown as mean ± SEM.
(TIF)

S4 Fig Lung histology and IFNα expression after X-47 challenge. (A) Representative pictures from hematoxylin and eosin stained lung tissue sections from A20^AEC-KO^ and control wild-type (WT) littermate mice at different times p.i. Scale bar, 100 μm. (B) IFNα, IFNβ, and CXCL10 protein levels in BAL fluid of A20^AEC-KO^ mice and control littermates as determined by ELISA on different time points after sublethal challenge with X-47. Data represent mean ± SEM of at least 4 mice per group. Data are representative of 2 independent experiments.
(TIF)

S5 Fig Influenza A X-47 infection does not significantly increase club cell apoptosis in A20^AEC-KO^ mice. (A) DEVDase assay to quantify caspase-3 activity in tissue homogenates of lungs of A20^AEC-KO^ and control littermates (A20^WT^) at different days post infection (days p.i.) with 0.05 X LD_50_ of X47 virus. Data are shown as mean ± SEM of at least 4 mice per group (*p < 0.05; 2-way ANOVA). (B) Flow cytometric Annexin V staining of EpCAM^+^ lung epithelial cells on collagenase type IV and DNase I digested lung tissue of A20^AEC-KO^ or A20^WT^ mice infected with 0.05 X LD_50_ X-47 at day 8 post infection. (C) Representative pictures from TUNEL stained lung tissue sections from A20^AEC-KO^ and control wild-type (WT) littermate mice at different times p.i. Scale bar, 100 μm.
(TIF)

S6 Fig DC kinetics in MLN and peripheral adaptive immune responses are not altered in A20^AEC-KO^ mice. (A) Absolute numbers of CD11b^-^, CD11b^+^ and inflammatory DCs (iDC) in mediastinal lymph nodes (MLN) measured by flow cytometry at 2, 5, 8 and 12 days post-infection (days p.i.) after challenge with 0.05 X LD_50_ X-47. (B) Cells isolated from lungs or spleens at day 8 p.i. were stimulated with indicated amounts of NP peptide (ASNENMETM). After 18h, brefeldin A was added for 6h and IFNγ expressing (IFNγ^+^) activated (CD62L^lo^) CD8^+^ T cells were analyzed using flow cytometry. (C) Virus specific antibody titers in serum at 12 and 20 days p.i. as determined by hemagglutination inhibition (HAI) assay (ND = not detected). Data show the results of 1 (A and B) or 2 (C) independent experiments and were analyzed using Student’s *t*-test (*p < 0.05) and are represented as mean ± SEM.
(TIF)

S7 Fig Reduced CCL2 expression in CCSP expressing club cells from A20^AEC-KO^ mice after influenza A virus infection. (A) CCL2 (MCP-1), KC (CXCL1) and Rantes (CCL5) protein levels in BAL fluid measured by Multiplex immunoassay at indicated time points post-infection. (B) Immunohistological section of inflamed A20^WT^ and A20^AEC-KO^ lung issue stained with anti-CCL2 (white) and anti-CCSP (red) at day 7 post-infection. Cell nuclei are staining with DAPI. Scale bar upper panels 50 μm, lower panels 10 μm.
(TIF)

## References

[1] Medzhitov R, Schneider DS, Soares MP (2012) Disease tolerance as a defense strategy. Science 335: 936–941. 10.1126/science.1214935 22363001 PMC3564547

[2] Soares MP, Gozzelino R, Weis S (2014) Tissue damage control in disease tolerance. Trends Immunol 35: 483–494. 10.1016/j.it.2014.08.001 25182198

[3] Baskin CR, Bielefeldt-Ohmann H, Tumpey TM, Sabourin PJ, Long JP, et al (2009) Early and sustained innate immune response defines pathology and death in nonhuman primates infected by highly pathogenic influenza virus. Proc Natl Acad Sci U S A 106: 3455–3460. 10.1073/pnas.0813234106 19218453 PMC2642661

[4] Cilloniz C, Shinya K, Peng X, Korth MJ, Proll SC, et al (2009) Lethal influenza virus infection in macaques is associated with early dysregulation of inflammatory related genes. PLoS Pathog 5: e1000604 10.1371/journal.ppat.1000604 19798428 PMC2745659

[5] Hillaire ML, Rimmelzwaan GF, Kreijtz JH (2013) Clearance of influenza virus infections by T cells: risk of collateral damage? Curr Opin Virol 3: 430–437. 10.1016/j.coviro.2013.05.002 23721864

[6] Sun J, Braciale TJ (2013) Role of T cell immunity in recovery from influenza virus infection. Curr Opin Virol 3: 425–429. 10.1016/j.coviro.2013.05.001 23721865 PMC3804899

[7] Iwasaki A, Pillai PS (2014) Innate immunity to influenza virus infection. Nat Rev Immunol 14: 315–328. 10.1038/nri3665 24762827 PMC4104278

[8] Rehwinkel J, Tan CP, Goubau D, Schulz O, Pichlmair A, et al (2010) RIG-I detects viral genomic RNA during negative-strand RNA virus infection. Cell 140: 397–408. 10.1016/j.cell.2010.01.020 20144762

[9] Baum A, Sachidanandam R, Garcia-Sastre A (2010) Preference of RIG-I for short viral RNA molecules in infected cells revealed by next-generation sequencing. Proc Natl Acad Sci U S A 107: 16303–16308. 10.1073/pnas.1005077107 20805493 PMC2941304

[10] Krug RM (2015) Functions of the influenza A virus NS1 protein in antiviral defense. Curr Opin Virol 12: 1–6. 10.1016/j.coviro.2015.01.007 25638592 PMC4470714

[11] Lund JM, Alexopoulou L, Sato A, Karow M, Adams NC, et al (2004) Recognition of single-stranded RNA viruses by Toll-like receptor 7. Proc Natl Acad Sci U S A 101: 5598–5603. 15034168 10.1073/pnas.0400937101 PMC397437

[12] Diebold SS, Kaisho T, Hemmi H, Akira S, Reis e Sousa C (2004) Innate antiviral responses by means of TLR7-mediated recognition of single-stranded RNA. Science 303: 1529–1531. 14976261 10.1126/science.1093616

[13] Le Goffic R, Pothlichet J, Vitour D, Fujita T, Meurs E, et al (2007) Cutting Edge: Influenza A virus activates TLR3-dependent inflammatory and RIG-I-dependent antiviral responses in human lung epithelial cells. J Immunol 178: 3368–3372. 17339430 10.4049/jimmunol.178.6.3368

[14] Le Goffic R, Balloy V, Lagranderie M, Alexopoulou L, Escriou N, et al (2006) Detrimental contribution of the Toll-like receptor (TLR)3 to influenza A virus-induced acute pneumonia. PLoS Pathog 2: e53 16789835 10.1371/journal.ppat.0020053 PMC1475659

[15] Leung YH, Nicholls JM, Ho CK, Sia SF, Mok CK, et al (2014) Highly pathogenic avian influenza A H5N1 and pandemic H1N1 virus infections have different phenotypes in Toll-like receptor 3 knockout mice. J Gen Virol 95: 1870–1879. 10.1099/vir.0.066258-0 24878639 PMC4135086

[16] Guillot L, Le Goffic R, Bloch S, Escriou N, Akira S, et al (2005) Involvement of toll-like receptor 3 in the immune response of lung epithelial cells to double-stranded RNA and influenza A virus. J Biol Chem 280: 5571–5580. 15579900 10.1074/jbc.M410592200

[17] Thomas PG, Dash P, Aldridge JR Jr., Ellebedy AH, Reynolds C, et al (2009) The intracellular sensor NLRP3 mediates key innate and healing responses to influenza A virus via the regulation of caspase-1. Immunity 30: 566–575. 10.1016/j.immuni.2009.02.006 19362023 PMC2765464

[18] Ichinohe T, Pang IK, Iwasaki A (2010) Influenza virus activates inflammasomes via its intracellular M2 ion channel. Nat Immunol 11: 404–410. 10.1038/ni.1861 20383149 PMC2857582

[19] Allen IC, Scull MA, Moore CB, Holl EK, McElvania-TeKippe E, et al (2009) The NLRP3 inflammasome mediates in vivo innate immunity to influenza A virus through recognition of viral RNA. Immunity 30: 556–565. 10.1016/j.immuni.2009.02.005 19362020 PMC2803103

[20] Parvatiyar K, Barber GN, Harhaj EW (2010) TAX1BP1 and A20 inhibit antiviral signaling by targeting TBK1-IKKi kinases. J Biol Chem 285: 14999–15009. 10.1074/jbc.M110.109819 20304918 PMC2865285

[21] Maelfait J, Roose K, Bogaert P, Sze M, Saelens X, et al (2012) A20 (Tnfaip3) deficiency in myeloid cells protects against influenza A virus infection. PLoS Pathog 8: e1002570 10.1371/journal.ppat.1002570 22396652 PMC3291650

[22] Vande Walle L, Van Opdenbosch N, Jacques P, Fossoul A, Verheugen E, et al (2014) Negative regulation of the NLRP3 inflammasome by A20 protects against arthritis. Nature 512: 69–73. 10.1038/nature13322 25043000 PMC4126806

[23] Tate MD, Schilter HC, Brooks AG, Reading PC (2011) Responses of mouse airway epithelial cells and alveolar macrophages to virulent and avirulent strains of influenza A virus. Viral Immunol 24: 77–88. 10.1089/vim.2010.0118 21449718

[24] Swamy M, Jamora C, Havran W, Hayday A (2010) Epithelial decision makers: in search of the 'epimmunome'. Nat Immunol 11: 656–665. 10.1038/ni.1905 20644571 PMC2950874

[25] Holt PG, Strickland DH, Wikstrom ME, Jahnsen FL (2008) Regulation of immunological homeostasis in the respiratory tract. Nat Rev Immunol 8: 142–152. 10.1038/nri2236 18204469

[26] Vareille M, Kieninger E, Edwards MR, Regamey N (2011) The airway epithelium: soldier in the fight against respiratory viruses. Clin Microbiol Rev 24: 210–229. 10.1128/CMR.00014-10 21233513 PMC3021210

[27] Heaton NS, Langlois RA, Sachs D, Lim JK, Palese P, et al (2014) Long-term survival of influenza virus infected club cells drives immunopathology. J Exp Med 211: 1707–1714. 10.1084/jem.20140488 25135297 PMC4144728

[28] Vereecke L, Sze M, Mc Guire C, Rogiers B, Chu Y, et al (2010) Enterocyte-specific A20 deficiency sensitizes to tumor necrosis factor-induced toxicity and experimental colitis. J Exp Med 207: 1513–1523. 10.1084/jem.20092474 20530205 PMC2901067

[29] Perl AK, Wert SE, Loudy DE, Shan Z, Blair PA, et al (2005) Conditional recombination reveals distinct subsets of epithelial cells in trachea, bronchi, and alveoli. Am J Respir Cell Mol Biol 33: 455–462. 16055670 10.1165/rcmb.2005-0180OC PMC2715353

[30] Perl AK, Zhang L, Whitsett JA (2009) Conditional expression of genes in the respiratory epithelium in transgenic mice: cautionary notes and toward building a better mouse trap. Am J Respir Cell Mol Biol 40: 1–3. 10.1165/rcmb.2008-0011ED 19075182 PMC2720111

[31] Winkelmann A, Noack T (2010) The Clara cell: a "Third Reich eponym"? Eur Respir J 36: 722–727. 10.1183/09031936.00146609 20223917

[32] Rawlins EL, Okubo T, Xue Y, Brass DM, Auten RL, et al (2009) The role of Scgb1a1+ Clara cells in the long-term maintenance and repair of lung airway, but not alveolar, epithelium. Cell Stem Cell 4: 525–534. 10.1016/j.stem.2009.04.002 19497281 PMC2730729

[33] Gon Y, Asai Y, Hashimoto S, Mizumura K, Jibiki I, et al (2004) A20 inhibits toll-like receptor 2- and 4-mediated interleukin-8 synthesis in airway epithelial cells. Am J Respir Cell Mol Biol 31: 330–336. 15142865 10.1165/rcmb.2003-0438OC

[34] Catrysse L, Vereecke L, Beyaert R, van Loo G (2014) A20 in inflammation and autoimmunity. Trends Immunol 35: 22–31. 10.1016/j.it.2013.10.005 24246475

[35] Ma A, Malynn BA (2012) A20: linking a complex regulator of ubiquitylation to immunity and human disease. Nat Rev Immunol 12: 774–785. 10.1038/nri3313 23059429 PMC3582397

[36] Alexopoulou L, Holt AC, Medzhitov R, Flavell RA (2001) Recognition of double-stranded RNA and activation of NF-kappaB by Toll-like receptor 3. Nature 413: 732–738. 11607032 10.1038/35099560

[37] Kato H, Takeuchi O, Mikamo-Satoh E, Hirai R, Kawai T, et al (2008) Length-dependent recognition of double-stranded ribonucleic acids by retinoic acid-inducible gene-I and melanoma differentiation-associated gene 5. J Exp Med 205: 1601–1610. 10.1084/jem.20080091 18591409 PMC2442638

[38] Ibanez LI, Roose K, De Filette M, Schotsaert M, De Sloovere J, et al (2013) M2e-displaying virus-like particles with associated RNA promote T helper 1 type adaptive immunity against influenza A. PLoS One 8: e59081 10.1371/journal.pone.0059081 23527091 PMC3601086

[39] Small BA, Dressel SA, Lawrence CW, Drake DR 3rd, Stoler MH, et al (2001) CD8(+) T cell-mediated injury in vivo progresses in the absence of effector T cells. J Exp Med 194: 1835–1846. 11748284 10.1084/jem.194.12.1835 PMC2193585

[40] Enelow RI, Mohammed AZ, Stoler MH, Liu AN, Young JS, et al (1998) Structural and functional consequences of alveolar cell recognition by CD8(+) T lymphocytes in experimental lung disease. J Clin Invest 102: 1653–1661. 9802879 10.1172/JCI4174 PMC509113

[41] Sun J, Madan R, Karp CL, Braciale TJ (2009) Effector T cells control lung inflammation during acute influenza virus infection by producing IL-10. Nat Med 15: 277–284. 10.1038/nm.1929 19234462 PMC2693210

[42] Guilliams M, Lambrecht BN, Hammad H (2013) Division of labor between lung dendritic cells and macrophages in the defense against pulmonary infections. Mucosal Immunol 6: 464–473. 10.1038/mi.2013.14 23549447

[43] Maus U, von Grote K, Kuziel WA, Mack M, Miller EJ, et al (2002) The role of CC chemokine receptor 2 in alveolar monocyte and neutrophil immigration in intact mice. Am J Respir Crit Care Med 166: 268–273. 12153956 10.1164/rccm.2112012

[44] Herold S, von Wulffen W, Steinmueller M, Pleschka S, Kuziel WA, et al (2006) Alveolar epithelial cells direct monocyte transepithelial migration upon influenza virus infection: impact of chemokines and adhesion molecules. J Immunol 177: 1817–1824. 16849492 10.4049/jimmunol.177.3.1817

[45] Narasaraju T, Ng HH, Phoon MC, Chow VT (2010) MCP-1 antibody treatment enhances damage and impedes repair of the alveolar epithelium in influenza pneumonitis. Am J Respir Cell Mol Biol 42: 732–743. 10.1165/rcmb.2008-0423OC 19617401 PMC2891499

[46] Lee EG, Boone DL, Chai S, Libby SL, Chien M, et al (2000) Failure to regulate TNF-induced NF-kappaB and cell death responses in A20-deficient mice. Science 289: 2350–2354. 11009421 10.1126/science.289.5488.2350 PMC3582399

[47] Turer EE, Tavares RM, Mortier E, Hitotsumatsu O, Advincula R, et al (2008) Homeostatic MyD88-dependent signals cause lethal inflamMation in the absence of A20. J Exp Med 205: 451–464. 10.1084/jem.20071108 18268035 PMC2271029

[48] Kallfass C, Lienenklaus S, Weiss S, Staeheli P (2013) Visualizing the beta interferon response in mice during infection with influenza A viruses expressing or lacking nonstructural protein 1. J Virol 87: 6925–6930. 10.1128/JVI.00283-13 23576514 PMC3676098

[49] Parvatiyar K, Harhaj EW (2011) Regulation of inflammatory and antiviral signaling by A20. Microbes Infect 13: 209–215. 10.1016/j.micinf.2010.11.003 21111841 PMC3031767

[50] Kok WL, Denney L, Benam K, Cole S, Clelland C, et al (2012) Pivotal Advance: Invariant NKT cells reduce accumulation of inflammatory monocytes in the lungs and decrease immune-pathology during severe influenza A virus infection. J Leukoc Biol 91: 357–368. 10.1189/jlb.0411184 22003207

[51] Zhou J, Wang D, Gao R, Zhao B, Song J, et al (2013) Biological features of novel avian influenza A (H7N9) virus. Nature 499: 500–503. 10.1038/nature12379 23823727

[52] Lin KL, Suzuki Y, Nakano H, Ramsburg E, Gunn MD (2008) CCR2+ monocyte-derived dendritic cells and exudate macrophages produce influenza-induced pulmonary immune pathology and mortality. J Immunol 180: 2562–2572. 18250467 10.4049/jimmunol.180.4.2562

[53] Herold S, Steinmueller M, von Wulffen W, Cakarova L, Pinto R, et al (2008) Lung epithelial apoptosis in influenza virus pneumonia: the role of macrophage-expressed TNF-related apoptosis-inducing ligand. J Exp Med 205: 3065–3077. 10.1084/jem.20080201 19064696 PMC2605231

[54] Ueda A, Okuda K, Ohno S, Shirai A, Igarashi T, et al (1994) NF-kappa B and Sp1 regulate transcription of the human monocyte chemoattractant protein-1 gene. J Immunol 153: 2052–2063. 8051410

[55] Valente AJ, Xie JF, Abramova MA, Wenzel UO, Abboud HE, et al (1998) A complex element regulates IFN-gamma-stimulated monocyte chemoattractant protein-1 gene transcription. J Immunol 161: 3719–3728. 9759897

[56] Aldridge JR Jr., Moseley CE, Boltz DA, Negovetich NJ, Reynolds C, et al (2009) TNF/iNOS-producing dendritic cells are the necessary evil of lethal influenza virus infection. Proc Natl Acad Sci U S A 106: 5306–5311. 10.1073/pnas.0900655106 19279209 PMC2664048

[57] McGill J, Van Rooijen N, Legge KL (2010) IL-15 trans-presentation by pulmonary dendritic cells promotes effector CD8 T cell survival during influenza virus infection. J Exp Med 207: 521–534. 10.1084/jem.20091711 20212069 PMC2839152

[58] Schuijs MJ, Willart MA, Vergote K, Gras D, Deswarte K, et al (2015) Farm dust and endotoxin protect against allergy through A20 induction in lung epithelial cells. Science 349: 1106–1110. 10.1126/science.aac6623 26339029

[59] Reed LJ, Muench H (1938) A simple method of estimating fifty percent endpoints. Am J Hyg 27: 493–497.

[60] Oreffo VI, Morgan A, Richards RJ (1990) Isolation of Clara cells from the mouse lung. Environ Health Perspect 85: 51–64. 2200669 10.1289/ehp.85-1568317 PMC1568317

[61] Hufford MM, Kim TS, Sun J, Braciale TJ (2011) Antiviral CD8+ T cell effector activities in situ are regulated by target cell type. J Exp Med 208: 167–180. 10.1084/jem.20101850 21187318 PMC3023137

