# Supplementary figures and images for: A20 Deficiency in Lung Epithelial Cells Protects against Influenza A Virus Infection
Source: PLoS Pathog. 2016 Jan 27;12(1):e1005410. doi: 10.1371/journal.ppat.1005410 (PMC4731390; doi:10.1371/journal.ppat.1005410)

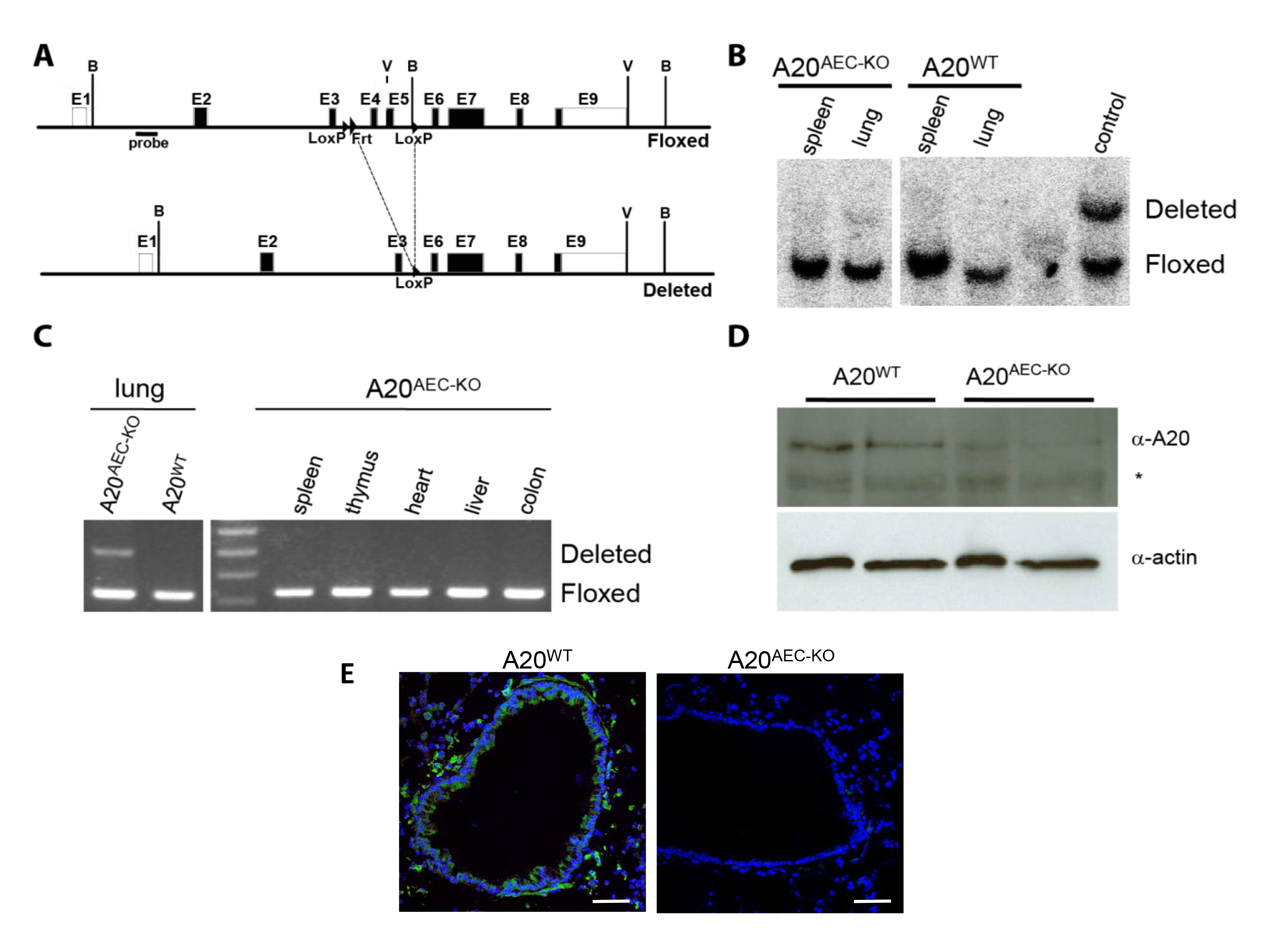

Supplement: S1 Fig — (A) Scheme of conditional A20 gene targeting. Boxes indicate exons 1–9 (E1-E9; black boxes depict coding sequence). E4 and E5 are flanked by 2 LoxP sites (arrowhead). Restriction enzymes (B, BamHI and V, EcoRV) and the location of the probe for Southern Blot analysis are also annotated. (B) Southern blot on genomic DNA isolated from spleens and lungs of A20AEC-KO or A20WT mice. DNA from heterozygous (A20+/-) MEFs was used as a control. (C) A20 specific PCR using primers that discriminate between floxed and deleted A20 alleles on genomic DNA isolated from lungs of A20WT or A20AEC-KO mice or DNA from different organs of A20AEC-KO mice. (D) Western blot for A20 and actin on club cells purified from lungs of A20WT or A20AEC-KO mice after intratracheal instillation of 1 μg LPS for 24 hours. (E) Immunohistological section of A20WT and A20AEC-KO lung tissue stained with anti-A20 antibody (green). Cell nuclei are staining with DAPI. Scale bar 20μm. (TIF) [file ppat.1005410.s001.tif]

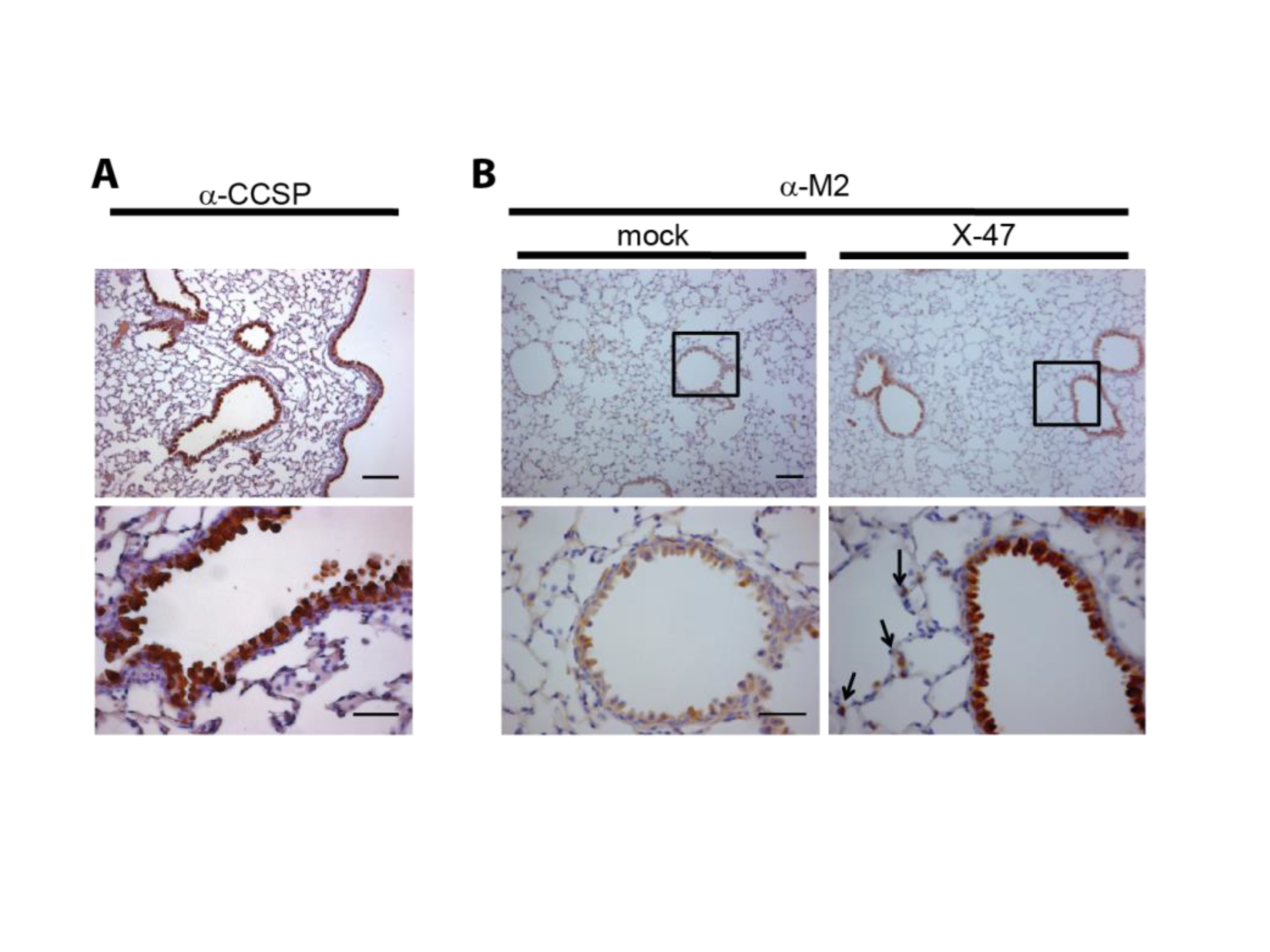

Supplement: S2 Fig — (A) Immunohistochemical stain for CCSP on lung sections of wild type mice (upper scale bar 50μm, lower scale bar 20μm). (B) Immunohistochemical stain for influenza A M2 protein on wild type mice mock (left) or X-47 virus infected (right) analyzed 4 days (4d) after infection (upper scale bar 50μm, lower scale bar 20μm). Lower panels represent magnifications of boxed sections of upper panels. Arrows indicate M2-positive alveolar epithelial cells. (TIF) [file ppat.1005410.s002.tif]

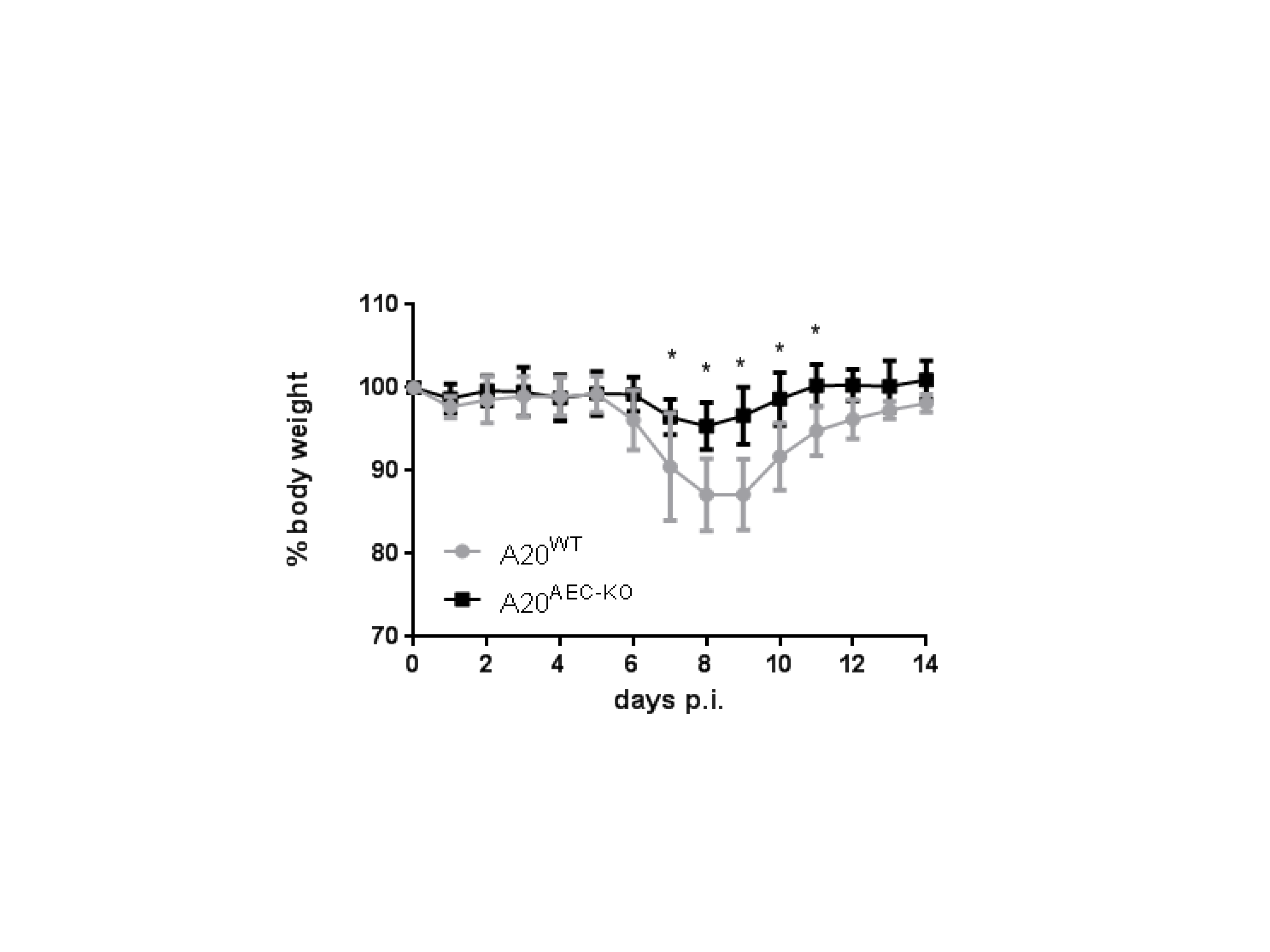

Supplement: S3 Fig — Weight loss of A20AEC-KO (n = 6) or wild type littermates (A20WT, n = 9) monitored until 14 days post infection (days p.i.) with a sublethal dose of the A/Puerto Rico/8/34 (PR8) strain (0.17 X LD50). Data were analysed using 2-way ANOVA (*p < 0.05) and are shown as mean ± SEM. (TIF) [file ppat.1005410.s003.tif]

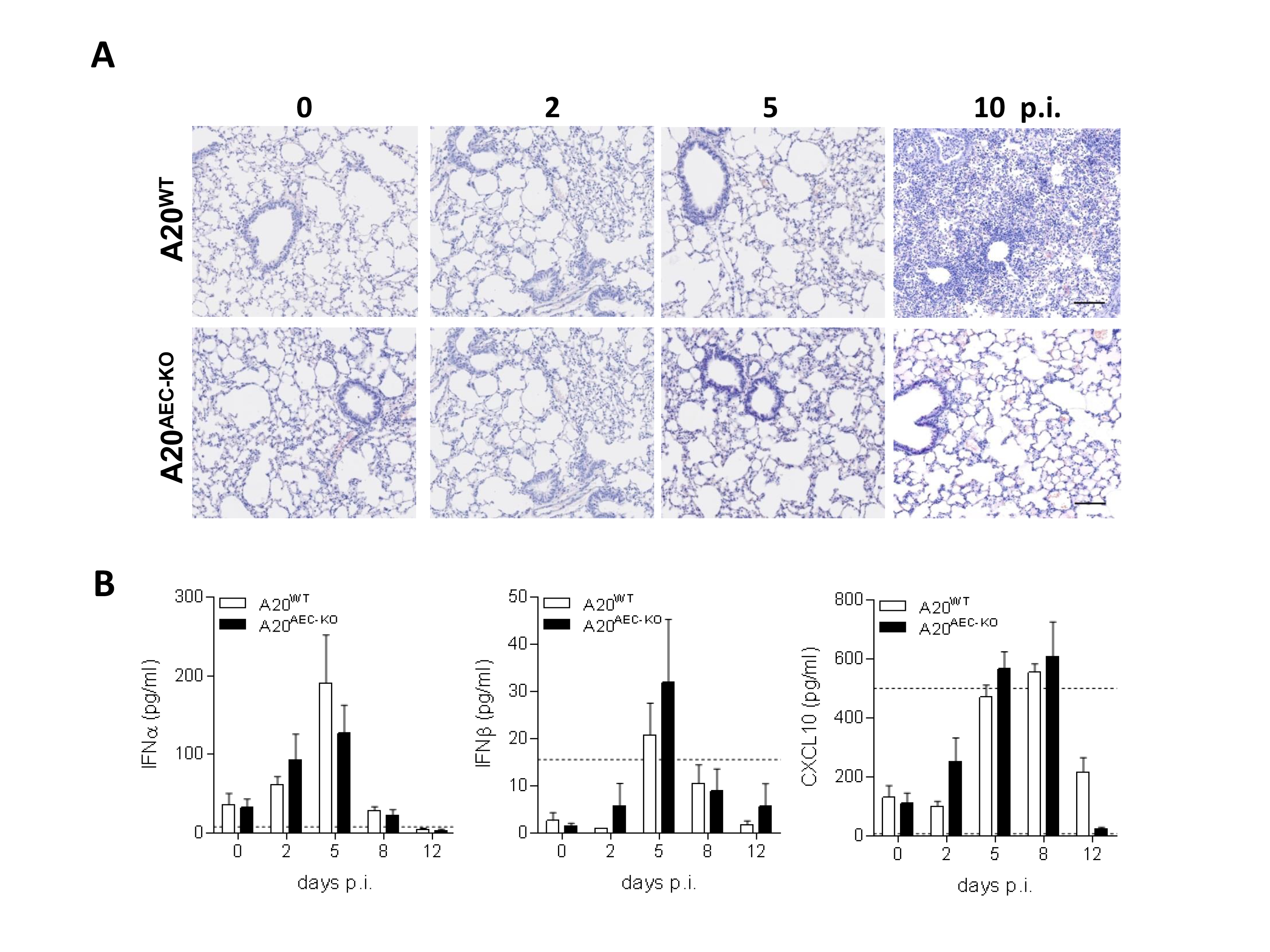

Supplement: S4 Fig — (A) Representative pictures from hematoxylin and eosin stained lung tissue sections from A20AEC-KO and control wild-type (WT) littermate mice at different times p.i. Scale bar, 100 μm. (B) IFNα, IFNβ, and CXCL10 protein levels in BAL fluid of A20AEC-KO mice and control littermates as determined by ELISA on different time points after sublethal challenge with X-47. Data represent mean ± SEM of at least 4 mice per group. Data are representative of 2 independent experiments. (TIF) [file ppat.1005410.s004.tif]

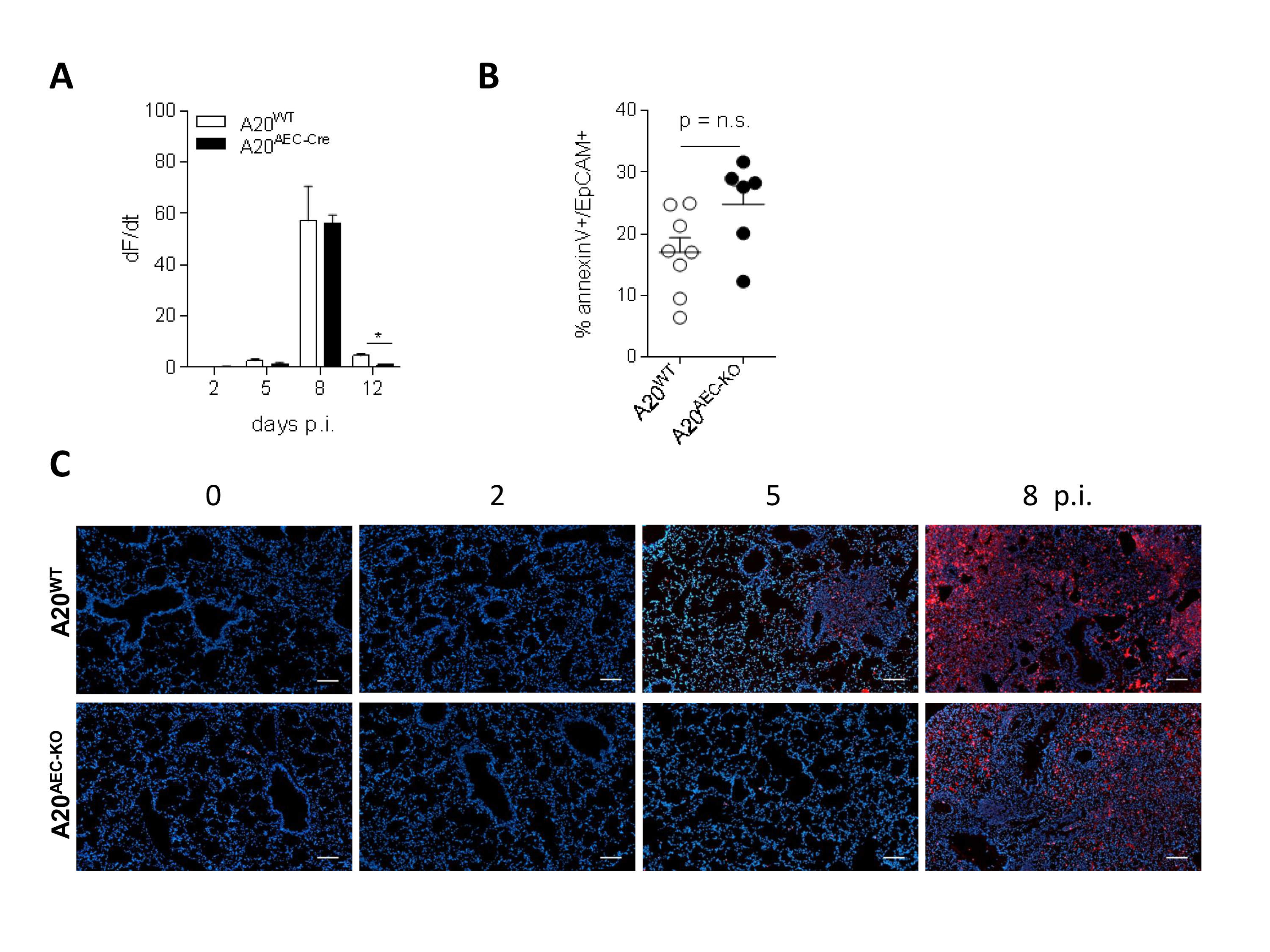

Supplement: S5 Fig — (A) DEVDase assay to quantify caspase-3 activity in tissue homogenates of lungs of A20AEC-KO and control littermates (A20WT) at different days post infection (days p.i.) with 0.05 X LD50 of X47 virus. Data are shown as mean ± SEM of at least 4 mice per group (*p < 0.05; 2-way ANOVA). (B) Flow cytometric Annexin V staining of EpCAM+ lung epithelial cells on collagenase type IV and DNase I digested lung tissue of A20AEC-KO or A20WT mice infected with 0.05 X LD50 X-47 at day 8 post infection. (C) Representative pictures from TUNEL stained lung tissue sections from A20AEC-KO and control wild-type (WT) littermate mice at different times p.i. Scale bar, 100 μm. (TIF) [file ppat.1005410.s005.tif]

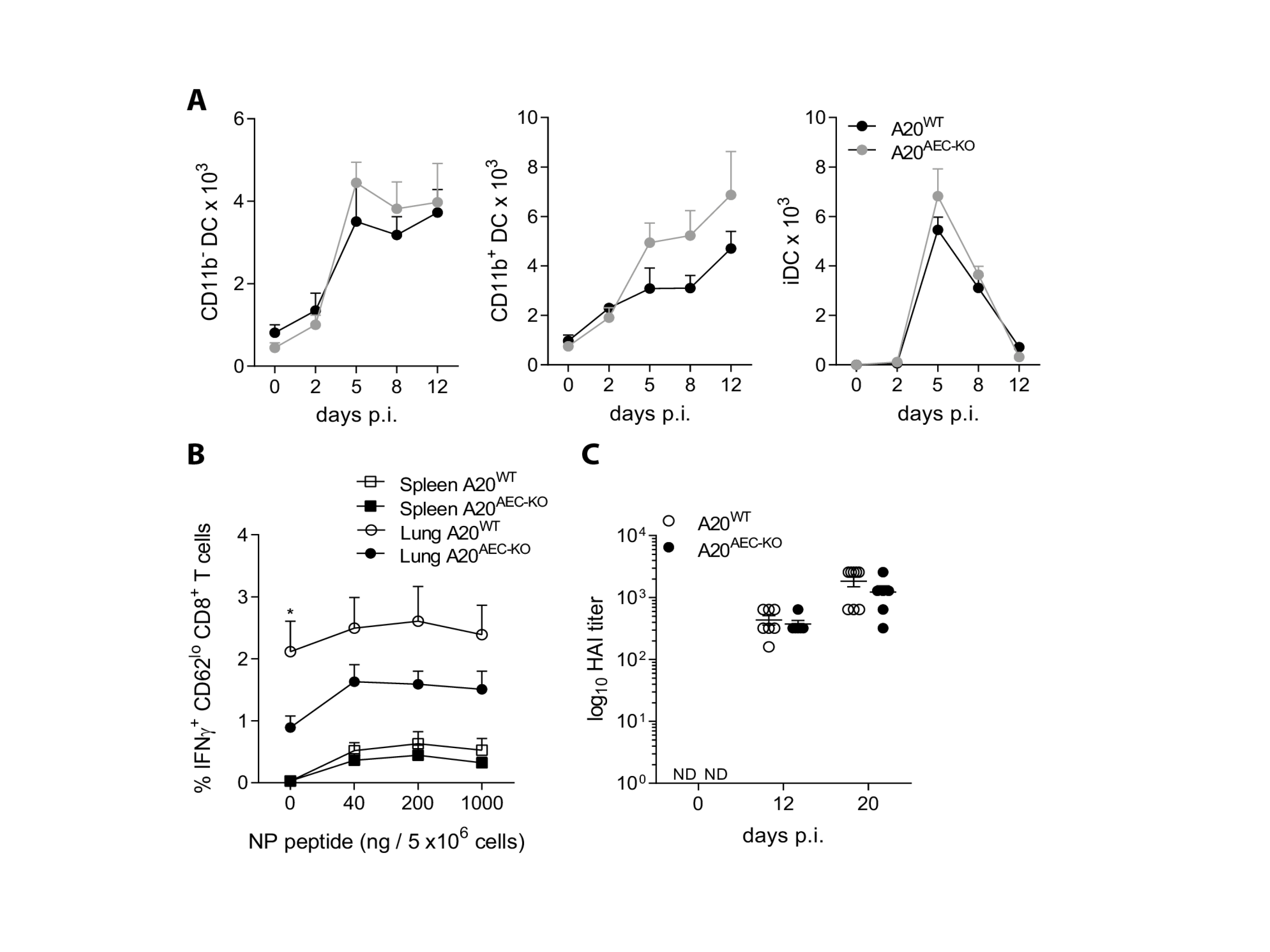

Supplement: S6 Fig — (A) Absolute numbers of CD11b-, CD11b+ and inflammatory DCs (iDC) in mediastinal lymph nodes (MLN) measured by flow cytometry at 2, 5, 8 and 12 days post-infection (days p.i.) after challenge with 0.05 X LD50 X-47. (B) Cells isolated from lungs or spleens at day 8 p.i. were stimulated with indicated amounts of NP peptide (ASNENMETM). After 18h, brefeldin A was added for 6h and IFNγ expressing (IFNγ+) activated (CD62Llo) CD8+ T cells were analyzed using flow cytometry. (C) Virus specific antibody titers in serum at 12 and 20 days p.i. as determined by hemagglutination inhibition (HAI) assay (ND = not detected). Data show the results of 1 (A and B) or 2 (C) independent experiments and were analyzed using Student’s t-test (*p < 0.05) and are represented as mean ± SEM. (TIF) [file ppat.1005410.s006.tif]

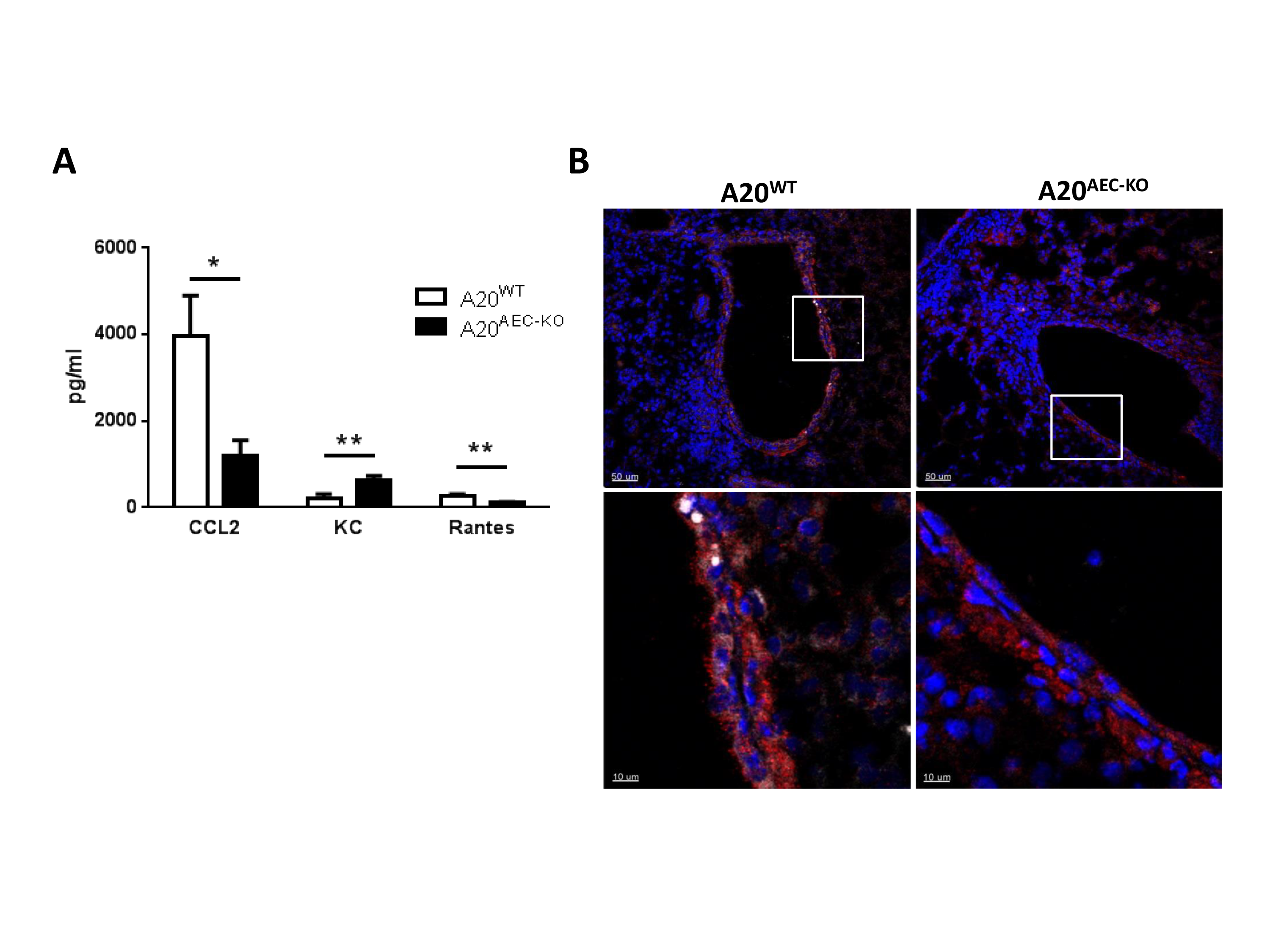

Supplement: S7 Fig — (A) CCL2 (MCP-1), KC (CXCL1) and Rantes (CCL5) protein levels in BAL fluid measured by Multiplex immunoassay at indicated time points post-infection. (B) Immunohistological section of inflamed A20WT and A20AEC-KO lung issue stained with anti-CCL2 (white) and anti-CCSP (red) at day 7 post-infection. Cell nuclei are staining with DAPI. Scale bar upper panels 50 μm, lower panels 10 μm. (TIF) [file ppat.1005410.s007.tif]
